# Intramedullary lengthening nails: can we also correct deformities?

**DOI:** 10.1007/s11832-016-0782-0

**Published:** 2016-11-15

**Authors:** U. Lenze, A. H. Krieg

**Affiliations:** University Children’s Hospital of Both Basel (UKBB), Spitalstrasse 31, 4031 Basel, Switzerland

**Keywords:** Mechanical axis, Axis deformity, Leg lengthening, Leg length discrepancy, Intramedullary nailing, Lengthening nail

## Abstract

Unlike external fixators, the use of solid intramedullary lengthening nails is restricted to defined anatomical preconditions, such as an adequate bone length. Furthermore, all deformity corrections except the lengthening procedure have to be implemented intraoperatively and cannot be adjusted postoperatively. Conversely, even complex deformity corrections can be performed using intramedullary devices after a thorough preoperative planning. For preparation of the intramedullary cavity as well as positioning of the lengthening nail according to the preoperative planning, reaming the medullary canal with rigid reamers which don’t follow the line of least resistance is inevitable. However, the application of solid lengthening nails might be limited, especially in children with ongoing epiphyseal growth, although a central perforation of the growth plate was shown to have no adverse effects on the growth potential. In cases with complex or multilevel deformities, an additional osteotomy and locking plate fixation could sometimes be a valuable solution in order to avoid external fixation. The low complication rate as well as the reduced compromising of soft tissues and periosteum render intramedullary lengthening nails the state-of-the-art procedure for limb lengthening in combination with deformity correction in patients who meet the anatomical preconditions.

## Historical overview of leg lengthening

One of the first pioneers of leg lengthening was Alesandro Codivilla who, in 1903, performed femoral osteotomies in patients with coxa vara by applying traction via a cast and a transcalcaneal wire [1]. About 10 years later, Louis Ombrédanne was the first to recognise the importance of a gradual lengthening and successfully performed a 4-cm femoral lengthening of 5 mm/day using an external fixator [2]. Another 10 years later, August Bier published his technique of delaying lengthening after the osteotomy [3]. The first ring fixator was introduced in the early 1950s by Wittmoser, but no attention was paid by his colleagues at that time [4]. The main breakthrough came with the observations of Gavril Ilizarov (1921–1992), a general practitioner in Kurgan (southwest Siberia, Russia) who treated countless numbers of war veterans for posttraumatic deformities, infected pseudarthroses, and bony defects [5]. He defined the main principles of leg lengthening and deformity correction such as the importance of a percutaneous corticotomy, a latency period of some days, a semi-rigid fixation and a defined distraction distance of 1 mm/day, which are still valid until today [5]. Although Ilizarov published his findings already in 1969, his techniques firstly attracted real attention about 12 years later, when he healed the long-standing pseudarthrosis of a well-known Italian journalist named Carlo Mauri [6, 7]. In the same year (1981), Ilizarov was invited to present his work at an AO infection conference in Lecco (Italy), initiating regular exchanges between Ilizarov and surgeons from the western world [7].

Another fundamental step was the introduction of intramedullary lengthening devices in the 1970s. The first systems, such as the hydraulic pressure-driven lengthening nail by Götz et al., were open-source systems with external components being directly linked to the nail and, therefore, having high failure rates due to deep infections [5, 8]. This handicap was overcome by fully implantable lengthening nails, the first being described in 1978 by Witt et al. [9]. Bliskunov developed a mechanically driven lengthening nail with a ratchet mechanism, in which length was generated via a hip movement-mediated compression of the nail clicker at the iliac wing [5]. In the last decade, especially two mechanical lengthening nails, namely, the Albizzia^®^ and the ISKD^®^ (Intramedullary Skeletal Kinetic Distractor), as well as the fully implantable motorised lengthening nail (Fitbone^®^), have been used consistently and described in the literature [10–12]. The latest developments of fully implantable lengthening nails are magnetically driven implants, namely, the Precice^®^ nail and the Phenix^®^ nail [13, 14].

## General considerations of intramedullary leg lengthening and deformity correction

Presumably due to lower complication rates and higher patient comfort, intramedullary lengthening devices have become an accepted alternative to external fixators [15–17]. Additionally, thanks to advances of intramedullary lengthening nails and the launch of new implants, the indications for their use have changed over the last decade, whereas even 3-dimensional deformity corrections are performed [5].

In contrast to external systems however, the use of straight solid intramedullary nails is subject to certain anatomical preconditions, such as an adequate bone length (according to the minimal implant length), suitable medullar dimensions (according to the minimal implant diameter) or the lack of marked angular deformities [15]. A centre of rotation and angulation (CORA) far from the planned osteotomy is an additional geometric obstacle, which might impair the use of intramedullary systems. Implant-associated restrictions may, furthermore, derive from a mandatory osteotomy level (certain min/max distance from the entry point) of some implants in order to achieve stable interlocking conditions [15, 17].

All deformity corrections except the lengthening procedure have to be implemented intraoperatively and—other than in external fixators and especially the Taylor spatial fame (TSF)—cannot be adjusted postoperatively. Furthermore, in contrast to lengthening with external fixation—which usually follows the mechanical axis—lengthening over a straight intramedullary nail occurs along the nail axis, which typically approaches the anatomical axis [18]. Thus, even in patients without angular deformities, changes of the mechanical axis are inevitable during the lengthening process (e.g. certain degree of valgisation in retrograde femur lengthening). If this geometry-caused axis shift is not taken into account preoperatively, intramedullary lengthening might result in an iatrogenic axis deformity [18]. Therefore, lengthening with/without concomitant angular deformities using straight implants always necessitates a thorough preoperative planning, as well as a thorough implantation technique [18, 19]. On the other hand, after preparing a meticulous preoperative planning, even complex deformity corrections are feasible with intramedullary lengthening devices when using straight rigid reamers (see below).

## Deformity correction with intramedullary nails

### Preoperative planning

**Fig. 1:**
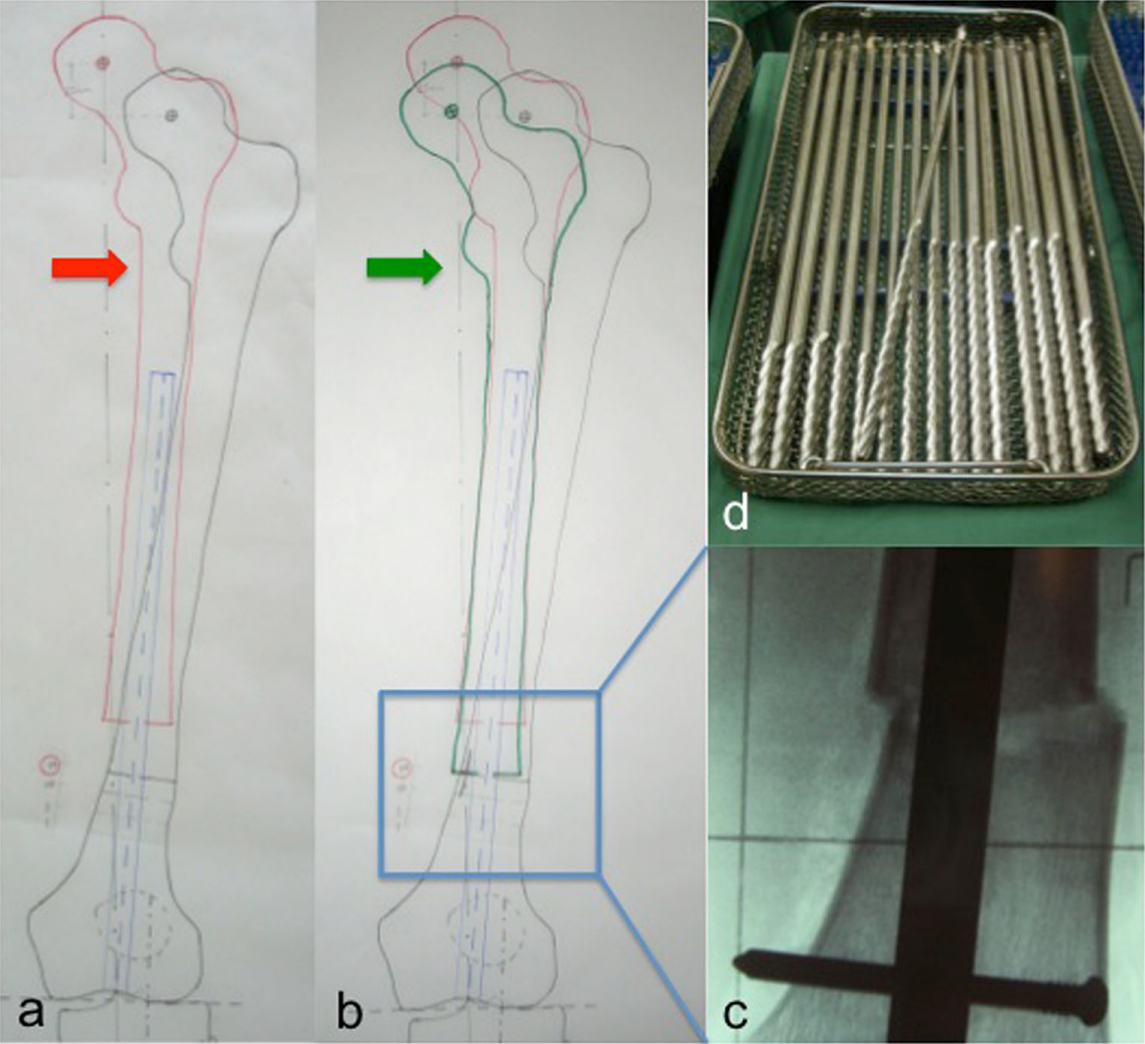
The principle of the reverse planning method [18] consists of planning in a first step the desired final result (*red arrow*) after deformity correction and lengthening (**a**). Afterwards, the lengthening procedure is graphically reversed (*green arrow*) and the corresponding implant position as well as the segment translation (*inset*) determined (**b**). The meticulous implementation of the preoperative planning (*inset* of **b**) is of utmost importance (**c**). Therefore, the use of straight rigid reamers is inevitable in order to prepare the medullary cavity and position the implant according to the preoperative planning (**d**)

The reverse planning method, which was introduced by Baumgart in 2009, is an ideal planning tool for leg lengthening and deformity correction using straight implants of both intramedullary lengthening nails or standard nails for fixator-assisted lengthening over nails [18]. Preoperatively, a standardised anteroposterior long standing radiograph (LSR) of both legs as well as a lateral view radiograph of the affected bone (including the neighbouring joints) is taken [18, 20]. The limb length discrepancy (LLD) as well as the preoperative mechanical axis alignment and projected joint angles such as the mechanical lateral distal femoral angle (mLDFA, physiological range 85°–90°) or the medial proximal tibial angle (MPTA, physiological range 85°–90°) have to be assessed, preoperatively [20]. Additionally, the joint range of motion at least of the affected leg should be examined. Depending on the intended approach (retrograde vs. antegrade) and the underlying deformity (frontal plane vs. sagittal plane etc.), a meticulous planning is performed according to Baumgart’s recommendations [18]. The principle of the reverse planning method is to simulate in a first step the desired final result after deformity correction and lengthening (Fig. 1a). Afterwards, the corresponding implant position of the expanded nail—which is necessary to achieve the simulated result—is graphically approximated and drawn on the planning. The entry point of the nail as well as the optimal osteotomy level are graphically defined too. Based on these measures, the lengthening process is virtually reversed along the assigned nail position (nail axis) and the appropriate degree of bone segment translation (which is necessary to realise the desired deformity correction) determined (Fig. 1a, b).

If a torsional malorientation is suspected (e.g. too big or too small lesser trochanter on anteroposterior LSR), an evaluation of the joint torsion by means of computed tomography (CT) or magnet resonance imaging (MRI) is recommended. Once the torsional malalignment has been objectified and is to be corrected during the surgery, an additional long standing radiograph (LSR 2) with external/internal rotation of the affected leg (according to the amount of torsional deformity measured in the CT) should be taken [18]. If the torsional deformity is not taken into account during the preoperative planning considerations but corrected intraoperatively, this might lead to axis deviations postoperatively. Therefore, in case of a femoral (torsional) deformity correction, the geometrical conditions of the lower leg up to the planned osteotomy level in the femur (extracted from LSR 1) have to be graphically combined with the geometrical conditions from the level of the osteotomy up to the femoral head in LSR 2 [18].

### Technical remarks

As the implant position cannot be adjusted postoperatively, a meticulous implementation of the preoperative planning is of utmost importance. For carrying out the anticipated correlations of the preoperative planning (implant position and corresponding bone translation after corticotomy), reaming with straight and rigid reamers (not flexible ones) as well as performing the osteotomy before reaming the second segment serve as a prerequisite [15–17] (Fig. 1c, d). Only rigid reamers, which don’t follow the line of least resistance, allow the preparation of the intramedullary cavity according to the desired implant position. Reamers with sharp tips are helpful to create new pathways by evenly removing cortical bone, whereas reamers with rounded tips are used to adjust the canal in a straight fashion [18]. The closer the osteotomy is performed to the joint line, the higher the amount of correction which can be achieved and the higher the amount of bone formation which is to be expected [19]. However, the closer the osteotomy is to the joint line, the less stability is achieved using intramedullary nails. In metaphyseal corrections, intramedullary lengthening was reported to be safe after acute angular deformity corrections of up to 30° [21]. The achieved mechanical axis alignment can be determined intraoperatively using a grid plate with radiopaque straight lines, which has to be placed underneath the patient preoperatively [15]. Especially in femoral corrections, it is recommended to place a 5-mm Schanz screw in the distal femur parallel to the joint line and a second one in the proximal femur in order to maintain control regarding the torsional orientation [18]. In cases with torsional deformity correction, the Schanz screws serve as hands for adjusting the right correction too.

### Indications and contraindications

A thorough and cautious determination of indications for intramedullary leg lengthening and deformity correction is inevitable. In our opinion, the indication for therapeutic leg lengthening consists of leg length discrepancies of 2.5 cm or more and especially patients in whom epiphysiodesis would not be a good option (premature growth plate, growth plate compromised by infection or tumour, short stature etc.). To our knowledge, detailed data regarding the indication for intramedullary lengthening in combination with deformity correction are lacking. However, the feasibility of the deformity correction using an intramedullary device should be simulated by means of an accurate preoperative planning in any case.

The application of solid lengthening nails in children might be limited by open growth plates. However, perforating the distal femoral growth plate through its central portion with a polished implant (e.g. retrograde nailing) was shown to have no adverse effects on the growth potential and to cause no iatrogenic deformities [18, 22]. Antegrade nailing of the tibia or femur, in contrast, is not recommended, as it might cause growth arrest. At our clinic, the use of intramedullary leg lengthening devices is waived during epiphyseal growth, except in justified individual cases. Otherwise, similar to patients with marked angular deformities which don’t meet the prerequisites for intramedullary nailing, the use of external fixators such as the TSF is recommended [15]. Further contraindications for intramedullary lengthening nails are—in our opinion—the evidence of osteomyelitis within the last 2 years or congenital deformities such as hip dysplasia.

Generally speaking, intramedullary limb lengthening in combination with deformity correction is demanding with respect to preoperative planning, operative technique and postoperative management and should, therefore, be reserved for experienced surgeons.

### Complex and multilevel deformities

**Fig. 2:**
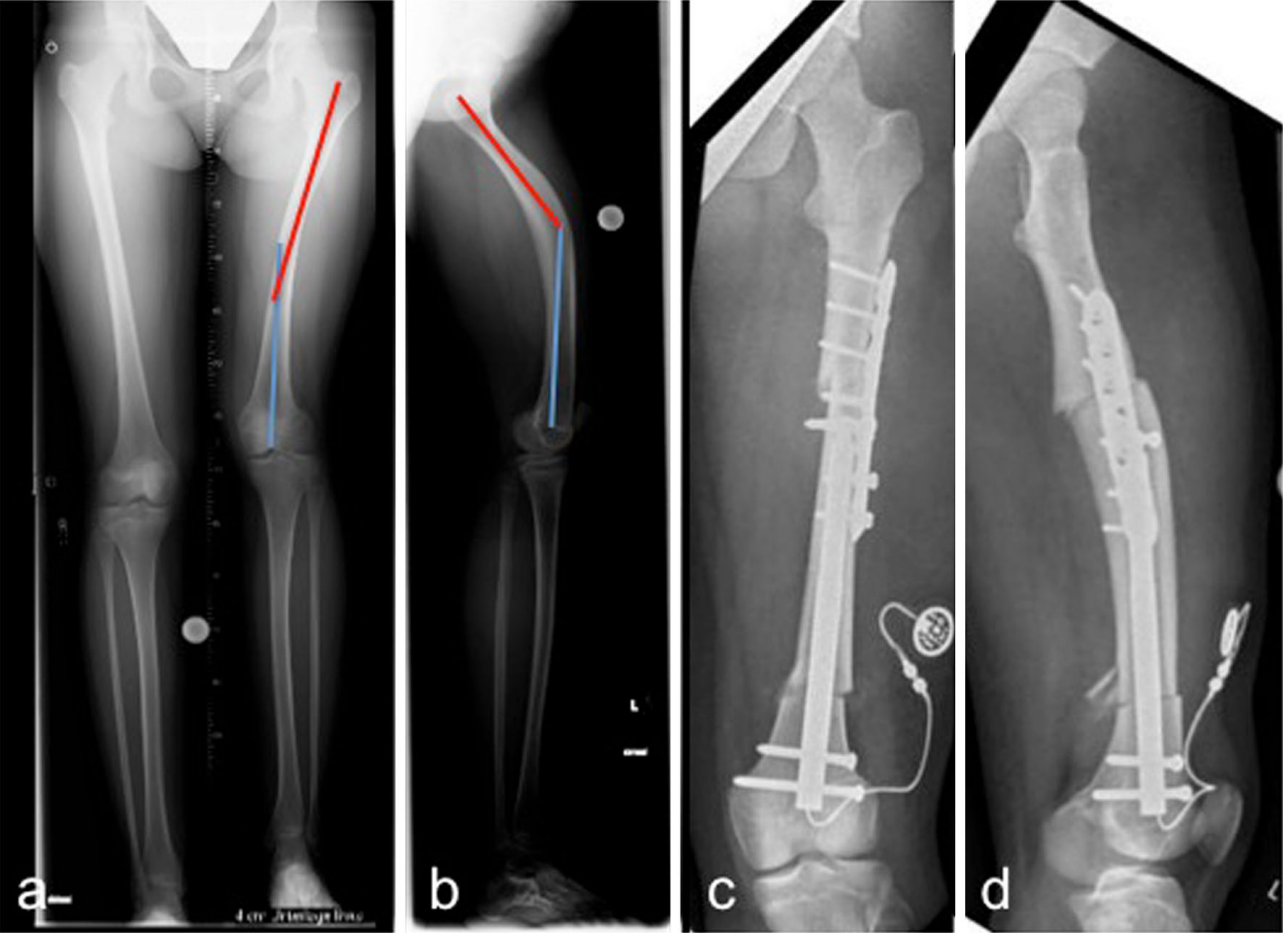
Preoperative anteroposterior (**a**) and laterolateral (**b**) long standing radiograph (LSR) of a 15-year-old patient with a combined valgus/flexion deformity and a leg length discrepancy of 4 cm. Respecting the patient’s desire, the use of an external fixator for deformity correction was avoided by performing a second osteotomy at the femoral diaphysis with additional plate fixation. Leg length equalisation was achieved by using the fully implantable motorised lengthening nail (Fitbone^®^) and a lengthening osteotomy at the distal femur. Postoperative result on anteroposterior (**c**) and laterolateral (**d**) X-rays

**Fig. 3:**
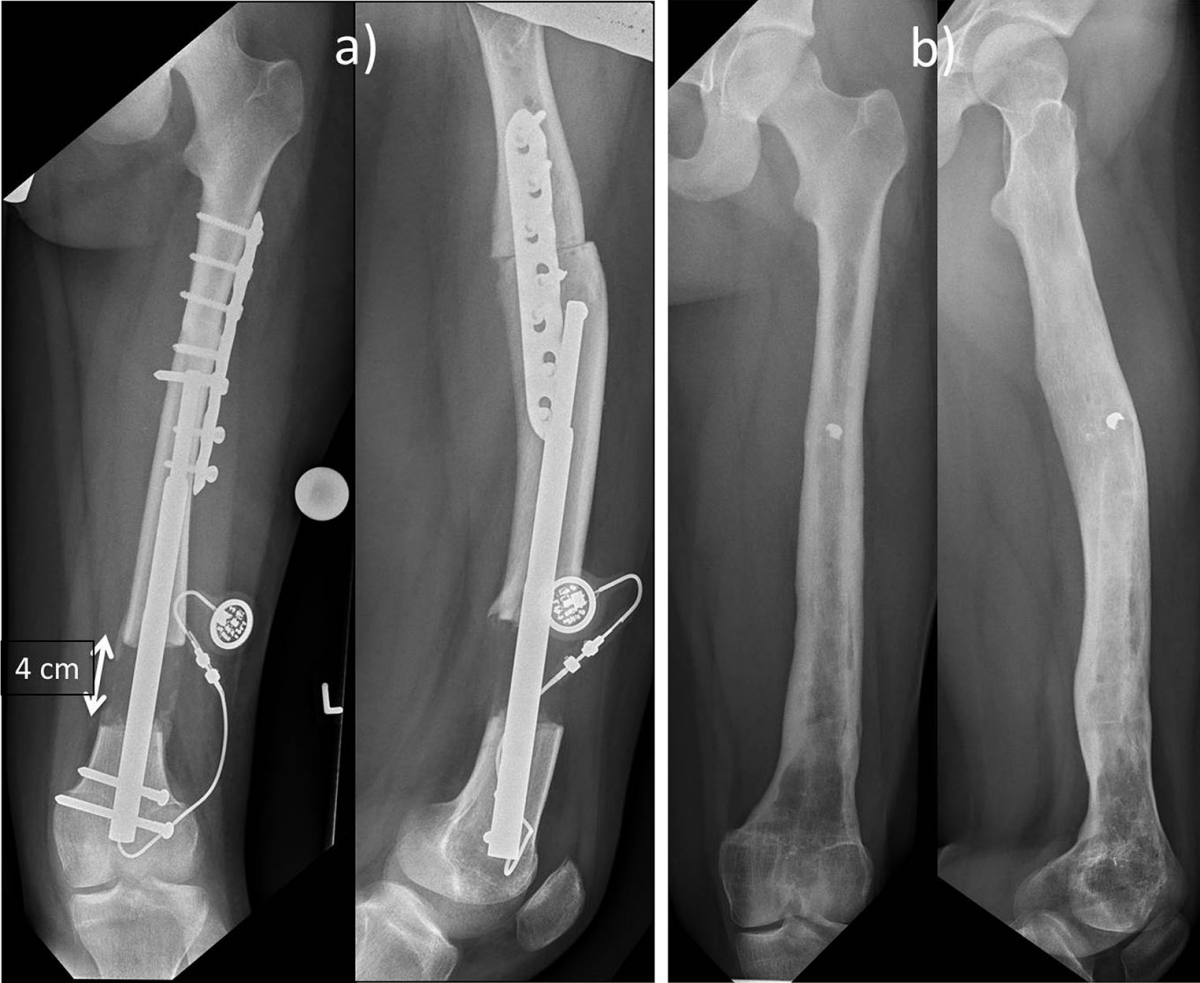
Anteroposterior and lateral X-rays after the distraction lengthening of 4 cm (**a**) and after removal of the metal 1.5 years later (**b**)

Anatomical conditions such as a CORA close to the joint line, multilevel deformities or a long sectional bending of the affected bone might complicate intramedullary deformity correction or even make it impossible. However, an additional osteotomy and locking plate fixation could be a valuable solution in selected cases in order to avoid the use of external fixators and typically fixator-associated complications (Figs. 2 and 3).

## Conclusion

Intramedullary lengthening nails are an accepted alternative to external fixators but are limited by anatomical preconditions. However, due to advances in preoperative planning methods and operative techniques, even complex deformities can sometimes be addressed with these implants. The use of rigid reamers as well as a meticulous implementation of the preoperative planning are of utmost importance. A second osteotomy and locking plate fixation might help to avoid the use of external fixators, even in cases with multilevel deformities. Nevertheless, in patients with marked angular deformities or open growth plates, the use of external fixators such as the Taylor spatial fame (TSF) is sometimes useful or even inevitable. However, in patients who meet the anatomical prerequisites, intramedullary lengthening nails is the state-of-the-art procedure for limb lengthening in combination with deformity correction due to low complication rates and high patient comfort.
